# Physical Factors Influencing Pleasant Touch during Passive Fingertip Stimulation

**DOI:** 10.1371/journal.pone.0101361

**Published:** 2014-07-07

**Authors:** Anne Klöcker, Calogero Maria Oddo, Domenico Camboni, Massimo Penta, Jean-Louis Thonnard

**Affiliations:** 1 Institute of Neuroscience, Université catholique de Louvain, Brussels, Belgium; 2 The BioRobotics Institute, Scuola Superiore Sant'Anna, Pisa, Italy; 3 Cliniques Universitaires Saint-Luc, Physical and Rehabilitation Medicine Department, Université catholique de Louvain, Brussels, Belgium; McMaster University, Canada

## Abstract

**Objective:**

Tactile explorations with the fingertips provide information regarding the physical properties of surfaces and their relative pleasantness. Previously, we performed an investigation in the active touch domain and linked several surface properties (*i.e.* frictional force fluctuations and net friction) with their pleasantness levels. The aim of the present study was to investigate physical factors being important for pleasantness perception during passive fingertip stimulation. Specifically we were interested to see whether factors, such as surfaces' topographies or their frictional characteristics could influence pleasantness. Furthermore, we ascertained how the stimulus pleasantness level was impacted by (i) the normal force of stimulus application (*F_N_*) and (ii) the stimulus temperature (*T_S_*).

**Methods and Results:**

The right index fingertips of 22 blindfolded participants were stimulated using 27 different stimuli, which varied in average roughness (*Ra*) and *T_S_*. A 4-axis robot moved the stimuli horizontally under participants' fingertips with three levels of *F_N_*. The robot was equipped with force sensors, which recorded the *F_N_* and friction force (*F_T_*) during stimulation. Participants rated each stimulus according to a three-level pleasantness scale, as very pleasant (scored 0), pleasant (scored 1), or unpleasant (scored 2). These ordinal pleasantness ratings were logarithmically transformed into linear and unidimensional pleasantness measures with the Rasch model. Statistical analyses were conducted to investigate a possible link between the stimulus properties (*i.e. Ra*, *F_N_*, *F_T_*, and *T_S_*) and their respective pleasantness levels. Only the mean *Ra* and *F_T_* values were negatively correlated with pleasantness. No significant correlation was detected between *F_N_* or *T_S_* and pleasantness.

**Conclusion:**

Pleasantness perception, resulting from passive fingertip stimulation, seems to be influenced by the surfaces' average roughness levels and average *F_T_* occurring during fingertip stimulation.

## Introduction

In everyday life, we continuously explore surfaces with our fingertips. These explorations provide information regarding the physical attributes of a surface (*e.g.* topography, frictional surface properties, and temperature), and are regularly accompanied by a perception of pleasantness.

The *physical parameters* of contact surfaces are perceived via the stimulation of various receptors embedded in the glabrous (*i.e.* non-hairy) skin [1]–[8]. They are all innervated through myelinated fibres (Aβ). Slowly adapting types I (SAI) and type II (SAII) afferents respond to a sustained stimulus with a sustained discharge. Rapidly adapting type I (RAI) and type II (RAII or PC) respond to dynamic changes of mechanical stimulation [1]. Each of these receptors has specific end organs and has been described as being implicated in the sensation of certain tactile inputs, such as tactile spatial acuity, the detection of skin stretch, roughness, or vibrations applied to the skin [1]–[7]. Type I receptors are surface-located and have a sharp and small receptive field, whereas type II receptors are deeply located and have a blurred and large receptive field [1]. Relating to the *pleasantness perception*, C-Tactile nerve fibres (CT-fibres) play a fundamental role in the detection and transmission of pleasant stimuli applied to hairy skin [9]–[12]. However, CT-fibres are missing from glabrous skin sites [13]–[15]; thus, mechanisms underlying the perception and transmission of the pleasantness of a tactile interaction remains unclear. One hypothesis is that the pleasantness of a surface is linked to its physical characteristics, which activate receptors in the fingertips. If this is the case, then it should be possible to link a surface's pleasantness level with its corresponding physical characteristics. Indeed, previous studies have shown that certain physical parameters of surfaces, such as topography, roughness, and temperature, may influence the perception of pleasantness. For instance, the subjective sensation of smoothness or roughness has been associated with a pleasant [16]–[17] or unpleasant [18]–[21] perception, respectively, during active [18]–[20] and passive touch [21]. Only a few studies have investigated the link between innocuous thermal sensations and pleasantness perception. One such study [22] highlighted that the cortical areas that process the affective value of innocuous thermal stimuli are different from those that process the sensory properties (*e.g.* intensity). However, in that study, thermal stimuli were not applied to the fingertips.

The perception of pleasantness induced by a tactile exploration may be regarded as a latent variable, similar to pain or intelligence. Latent variables are typically estimated through indirect measurement methods (*e.g.* questionnaires) that generate ordinal data and exclude the possibility of applying parametric statistical methods. Probabilistic measurement models, such as the Rasch model [23], can be used to transform ordinal data into linear, unidimensional, and invariant measures (*see* Introduction S1 for more details on the Rasch model).

To build a solid and objective basis for investigating the perception of pleasantness elicited through surface explorations with the fingertips, we developed a unidimensional *Pleasant Touch Scale* through the Rasch model [24]. This scale classifies 37 common materials (*e.g.* sandpaper, wood, marble, fabrics, papers, etc.) according to the pleasantness level they elicit during *active* surface explorations with the fingertip. In line with previous studies [16]–[21], the results of this study indicated that the surface topography impacts pleasantness perception [24]. Furthermore, we observed that subjects' fingertip moisture levels influenced the perceived pleasantness of the explored surface [24]. This finding suggests that during active surface exploration, friction at finger-surface interface might be implicated in the pleasantness perception. A second study confirmed that surface topography and friction are crucial factors in pleasantness perception [25]. This latter study also highlighted that participants spontaneously chose a preferred normal force and exploration velocity, regardless of the surface being explored with their fingertips [25].

In the present study, we sought to extend our objective investigations in the area of pleasant touch perception at the fingertip level. Specifically, we investigated physical factors being implicated in pleasantness perception during *passive* fingertip stimulation, such as the stimulus surface topography (or average roughness levels). Furthermore we examined whether the pleasantness perception resulting from passive fingertip stimulation is affected by (i) the stimulus temperature and (ii) the applied normal force of the stimulus.

## Materials and Methods

### Participants

A total of 22 healthy subjects (10 males; age range 22–56 years) were enrolled in this study. The study was approved by the Biomedical Ethical Commission of the Faculty of Medicine of the Université Catholique de Louvain, Belgium (2010/07JUI/174, Belgian registration number: B40320108947). Participants provided their written informed consent to participate in this study. This consent procedure was approved by the ethics committee.

### Experimental Apparatus

**Figure 1:**
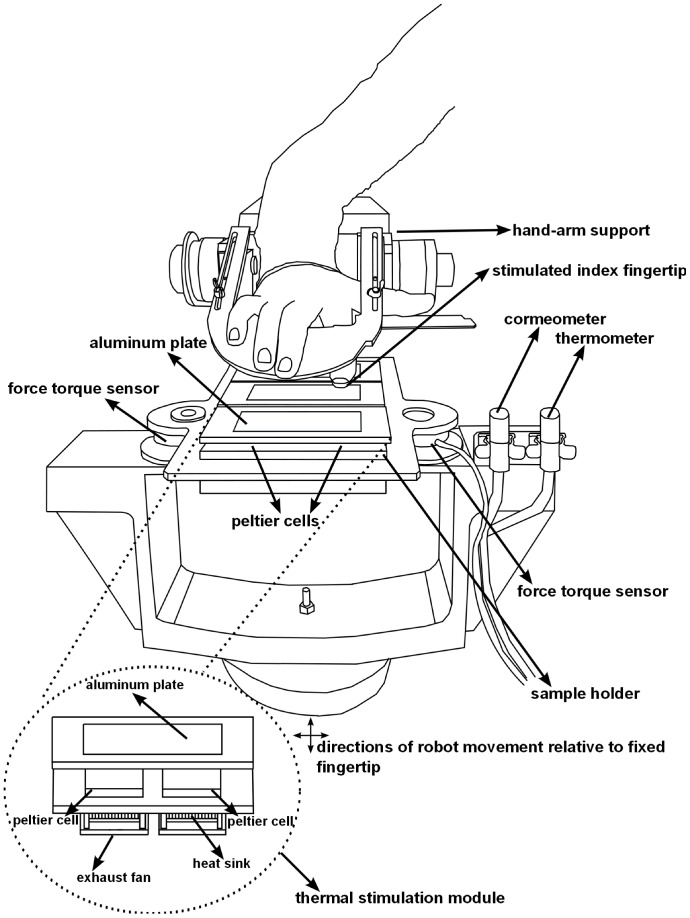
Illustration of the measurement experimental apparatus.

Figure 1 illustrates the experimental apparatus, which consisted of three thermal stimulation modules (TSMs). Every TSM allows the aluminium plate temperature to be regulated between 10°C and 50°C by using two high performance Peltier cells (HP-127-1.0-1.3-71P, TE Technology, Inc., Traverse City, MI, USA), two heat sinks (MBF35003-24W/2.6, Malico, Inc., Taiwan), and two exhaust fans (GM1203PFV1-8 F-GN, Sunonwealth Electric Machine Industry Co., Ltd., Taiwan) that help to remove excess heat from the Peltier cells. Two Negative Temperature Coefficient thermistors (TCS-610, Wavelength Electronics, Inc., Bozeman, MT, USA), embedded in the aluminium plates, and two linear proportional-integral temperature controllers (HTC3000, Wavelength Electronics, Inc.) allow measurement of the stimulus temperature (*T_S_*) and thermal feedback. A custom mounting system allows fast replacement of the aluminium plate on the top of the TSMs.

Three TSMs were rigidly fixed on an aluminium frame. This structure was installed on top of two 6-axis, strain-gauge force-torque sensors (Mini 40 and Nano 43, ATI Industrial Automation, Inc., Apex, NC, USA), which were positioned on a 4-axis robot (4-axis SCARA HS series 4535G, DENSO Products and Services Americas, Inc., CA, USA). The force sensors measure the linear forces in three dimensions, *i.e.* one force vector normal to the contact surface (*F_Z_*) and two force vectors tangential to the surface (*F_X_* and *F_Y_*), with a resolution of about 20 mN. The robot can be controlled in the normal, tangential, and rotational directions with predefined velocities. The force sensor signals, robot position, and *T_S_* were acquired at a sampling rate of 1 kHz.

A Corneometer CM 825 (CK electronic GmbH, Köln, Germany) was fixed on the measurement experimental apparatus and was used to measure the fingertip moisture level (*M*), room temperature (*T_R_*), and relative humidity (*H*) during the experiment. The fingertip temperature (*T_F_*) was measured through an infrared thermometer (Raytek MI3, Raytek Corporation, Santa Cruz, CA, USA), which was fixed on the apparatus. A hand-arm support on the apparatus allowed the participant to rest his or her arm and hand such that only the right index fingertip was stimulated (Figure 1).

**Figure 2:**
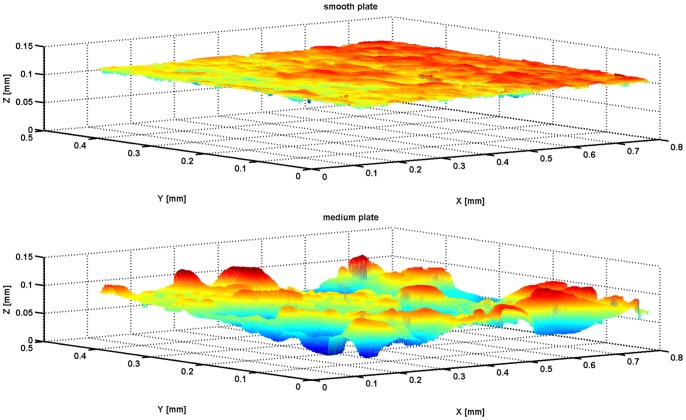
Three-dimensional illustration of aluminium plates characterized through white-light interferometry. The top part illustrates the smooth plate and the bottom part the medium plate.

Three aluminium plates with different average roughness (*Ra*) levels were obtained through controlled electric discharge machining of their surfaces. The *Ra* values were characterized by surface contact profilometry (Dektak 150 profiler, Veeco Instruments Inc., AZ, USA) and white-light interferometry (Polytek MSA-500, Polytec GmbH, Waldbronn, Germany). Three profilometry measures were taken per aluminium plate along its long axis (*i.e.* the stimulation direction). The mean *Ra* values were 1.4 ± 0.1 µm (smooth plate), 13.1 ± 1.1 µm (medium-roughness plate), and 40 ± 3 µm (rough plate). For additional verification of the surface characterization measures, interferometry was performed for the smooth and medium-roughness plates. The mean *Ra* values of the smooth (1.8 ± 0.3 µm) and medium-roughness (12.9 ± 3 µm) plates were similar to those measured through profilometry. Figure 2 illustrates the three-dimensional (3D) surfaces of the smooth (panel A) and medium-roughness (panel B) plates. All subsequent statistical analyses were based on the mean *Ra* values determined through profilometry surface characterization.

Each aluminium plate could be heated or cooled by fixation on the TSM of the measurement apparatus. Each plate was applied to the index fingertip with three levels each of normal force (*F_N_*  =  0.5, 1, and 2N) and *T_S_* (15°C, 30°C, and 40°C). Thus, 27 stimuli were used during the experiment (*i.e.* combination of three *Ra*, three *F_N_*, and three *T_S_*). The *F_N_* range was chosen on the basis of our previous observation [25] that healthy subjects spontaneously choose exploration forces in the range of 0.2 to 1.6N. The *T_S_* values were chosen to range from non-painfully cold (threshold ∼ 9–10°C) to non-painfully hot (threshold ∼43–47°C) [22]; [26]–[27].

### Experimental Procedure

Each participant was installed next to the experimental measurement apparatus. The right upper limb was comfortably positioned for stimulation (Figure 1). The subject was first habituated with the experimental procedure through a training session that was identical to the test session described below, except that only 10 stimuli were applied. Thereafter, the participant was blindfolded, and the values of *T_R_* and *H* were measured. Before fingertip stimulation, the robot was positioned to place the Corneometer CM 825 just beneath the participant's right index fingertip to measure *M*. The robot was repositioned to measure *T_F_*.

The robot was then positioned to place the aluminium plate underneath the subject's fingertip. The initial stimulation (*i.e.* application of one aluminium plate at one *F_N_* and *T_S_*) was applied in three phases. First, the robot was moved vertically to touch the index fingertip and apply the required *F_N_*. Second, the robot was maintained stationary in contact with the index fingertip for 5 seconds, to achieve a stable *F_N_* and to allow the subject to perceive the temperature. Third, the robot was moved horizontally (from right to left) at 35 mm/s to apply the stimulus to the participant's index fingertip. Participants were asked to pay special attention to the third phase to rate the pleasantness level of the stimulation. Each stimulus was rated with a three-level ordinal scale, as very pleasant (scored 0), pleasant (scored 1), or unpleasant (scored 2). The choice of this three-level ordinal scale was based on our past study [24] which highlighted that subjects performing a task comparable to the one required in the present study were not able to discriminate between “very unpleasant” and “unpleasant” categories.

**Figure 3:**
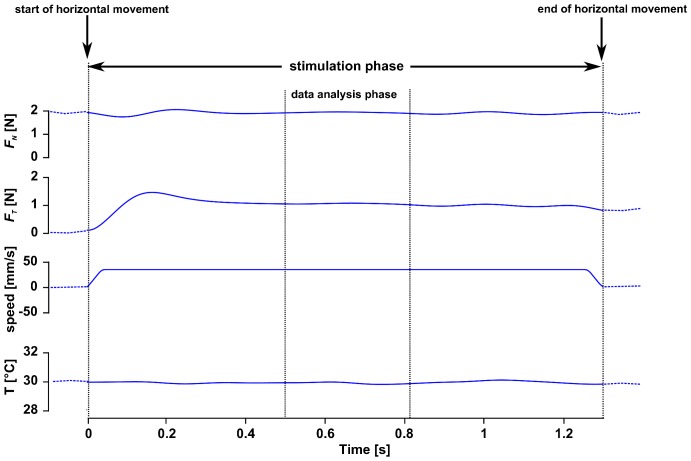
Illustration of a typical stimulation trial. Outermost vertical lines delimit the stimulation phase. Innermost vertical lines delimit the data analysis phase. The following variables are represented according to time in a top-down manner: normal force (*F_N_*), tangential force (*F_T_*), stimulation speed (speed), and stimulus temperature (T).

The same procedure (*i.e.* measurement of *M*, *T_F_*, and the three stimulation phases) was repeated for the remaining 26 stimuli. One block of 27 stimuli applied in random order lasted approximately 20 minutes. To control whether the participants' pleasantness ratings remained coherent, each participant was stimulated with three different blocks. Between two successive stimulation blocks, participants were allowed to remove the blindfold. During fingertip stimulation, the tangential force (*F_T_*) and *F_N_* components were recorded, along with *T_S_* and the stimulation position. Figure 3 illustrates a typical trial of the signals recorded during the stimulation phase.

When the aluminium plates were applied at 15°C, the mean *M* and *T_F_* were 39 ± 2.7 arbitrary units (a.u.) and 33°C ± 0.2°C, respectively. When the plates were applied at 30°C, *M* was 40 ± 3.2a.u. and *T_F_* was 32°C ± 1°C. When the plates were applied at 40°C, *M* was 39 ± 3.4a.u. and *T_F_* was 33°C ± 0.2°C. The *T_R_* ranged from 23.1°C to 30.9°C, and *H* ranged from 44.5% to 58.1%. Table 1 summarizes the mean *F_T_* and mean dynamic coefficient of friction (*μ*  =  *F_T_*/*F_N_*) per aluminium plate and *F_N_*.

**Table 1:** *F_T_* and *μ* data per aluminium plate and per *F_N_*.

Normal force [N]	Smooth plate	Medium-roughness plate	Rough plate
0.5	FT = 0.27 ± 0.12 [N]	FT = 0.22 ± 0.03 [N]	FT = 0.25 ± 0.01 [N]
	μ = 0.54 ± 0.24 [−]	μ = 0.44 ± 0.06 [−]	μ = 0.5 ± 0.02 [−]
1.0	FT = 0.52 ± 0.22 [N]	FT = 0.46 ± 0.03 [N]	FT = 0.56 ± 0.02 [N]
	μ = 0.52 ± 0.22 [−]	μ = 0.46 ± 0.03 [−]	μ = 0.56 ± 0.02 [−]
2.0	FT = 1.01 ± 0.42 [N]	FT = 0.92 ± 0.16 [N]	FT = 1.14 ± 0.01 [N]
	μ = 0.63 ± 0.22 [−]	μ = 0.45 ± 0.08 [−]	μ = 0.57 ± 0.007 [−]

Data are mean ± SD. *F_T_*: tangential force; *μ*: dynamic coefficient of friction.

### Data Processing

For each stimulus, non-parametric Friedman tests were conducted to test whether the participants' pleasantness ratings remained coherent during the three stimulation blocks. For each of these tests, the dependent variable was the ordinal pleasantness rating. The null hypothesis of each of these analyses was that the pleasantness ratings did not vary from one block to the next and effects were considered significant for p < 0.05. The results of these analyses highlighted that all p-values ranged between 0.1 and 0.97. Consequently, the null hypothesis could not be rejected, which demonstrated that the pleasantness ratings did not vary significantly from one block to the next one. Thus, the ordinal pleasantness ratings were considered coherent, and one stimulation block was randomly chosen per participant. Consequently, one ordinal total pleasantness score could be calculated per stimulus. Scores could range from 0 (all participants rated it very pleasant, 0 × 22) to 44 (all participants rated it unpleasant, 2 × 22).

As ordinal scores lack fundamental psychometric properties, they are not amenable to parametric statistics. To overcome this limitation, the Rasch model was used to transform the ordinal total scores logarithmically into linear and unidimensional pleasantness measures. The measurement unit of the scale is the logit. Lower logit values indicate less pleasant stimuli (*see* Introduction S1 for more details on the Rasch model). Through an invariance analysis, the Rasch model was used to investigate whether the pleasantness measures of the stimuli were significantly influenced by *M*, *T_F_*, *T_R_*, *H*, or participant age. All Rasch analyses were performed with RUMM (RUMM2020, RUMM Laboratory Pty Ltd., Perth, Western Australia) using the rating scale model.

Analyses regarding *F_T_*, *F_N_*, and *T_S_* focused on 600 ms of the steady-state fingertip stimulation phase (third phase) (Figure 3) of the same randomly chosen stimulation blocks, as for the Rasch analysis described above. The Matlab (version 7.10) software package was used for data processing. All data were numerically low-pass filtered with a Butterworth 4^th^ order filter at 5 Hz. The mean values of *F_T_*, *F_N_*, and *T_S_* were calculated per stimulation and per participant.

### Statistical Analyses

A stepwise forward multiple linear regression was conducted to investigate whether the pleasantness perception of the stimulation could be predicted by the (i) surface topography, (ii) friction force during stimulation, (iii) normal force with which the stimulus was applied, or (iv) stimulus temperature. Linear and unidimensional pleasantness measures were defined as dependent variables. Independent variables were *Ra*, *F_T_*, *F_N_*, *T_S_*, *T_F_*, and *M*. One mean value per variable was calculated per material. The forward model consists of first selecting the variable that best predicts the dependent variable (*i.e.* pleasantness). Then, the model adds the variable that accounts for the next largest prediction of pleasantness, and verifies whether the first variable remains a useful predictor. If this variable is no longer useful, then the model removes it. The procedure is repeated until the best model is defined.

All regression analyses were performed with JMP 10.0 (SAS Institute Inc., Cary, NC 27513, USA). Effects were considered significant for p < 0.05. To investigate whether *T_S_* or *Ra* had an effect on *μ*, IBM SPSS Statistics (version 20) was used to conduct repeated-measures analyses of variance (RM-ANOVA), in which *T_S_* and *Ra* were defined as “within-participant factors” and the “within-participant variable” was *μ*.

## Results

One stimulus was rated as unpleasant by every participant (rough plate at 2N and 15°C) and, thus, had an extreme score. This indicates that the stimulus was “too unpleasant” for the subject sample. It is not possible to determine a definite pleasantness level for such stimuli. Therefore, it was excluded from further investigations. The final *Passive Pleasant Touch Scale* was formed of the 26 remaining stimuli.

Table 2 presents each stimulus with its corresponding pleasantness measures (in logit) and standard error. The rough plate at 2N and 40°C was the most unpleasant stimulus of the scale, whereas the smooth plate at 0.5N and 30°C was the most pleasant one. The odds of observing any particular stimulus as pleasant rather than unpleasant increases by a factor of 2.71 (*i.e.* base of the natural logarithm, *e*) with each logit [28]. Pleasantness levels ranged from −4.3 to 2.3 logits (*i.e.* range of 6.6 logits). This means that, for any subject, the odds of rating the most pleasant stimulus as pleasant were e^6.6^  =  735 times higher than the odds of rating the least pleasant stimulus as pleasant. According to the results of the invariance analysis, the pleasantness levels of the 26 stimuli of the *Passive Pleasant Touch Scale* were not influenced by the age or gender of participants, *T_F_*, *M*, *T_R_*, or *H*.

**Table 2:** Stimulus pleasantness measures with the corresponding standard errors.

Stimulus	Pleasantness [logit]	SE [logit]
R F2 T40	−4.359	1.332
R F2 T30	−3.004	0.731
R F1 T40	−1.449	0.431
R F1 T15	−1.396	0.425
R F1 T30	−1.395	0.425
M F2 T15	−1.145	0.401
R F0.5 T15	−0.523	0.359
S F2 T15	−0.414	0.355
R F0.5 T30	−0.382	0.353
R F0.5 T40	−0.325	0.351
M F2 T40	−0.002	0.342
M F2 T30	0.123	0.339
S F1 T15	0.218	0.338
S F2 T40	0.231	0.338
S F0.5 T15	0.232	0.338
M F0.5 T15	0.35	0.337
M F1 T15	0.393	0.337
M F1 T40	0.572	0.337
M F1 T30	0.915	0.341
M F0.5 T40	1.012	0.343
S F1 T40	1.023	0.343
M F0.5 T30	1.400	0.356
S F2 T30	1.594	0.366
S F0.5 T40	1.962	0.390
S F1 T30	2.035	0.396
S F0.5 T30	2.336	0.424

*S*: smooth plate; *M*: medium-roughness plate; *R*: roughest plate; *F*: normal force in N; *T*: stimulus temperature in °C; *SE*: standard error.

**Figure 4:**
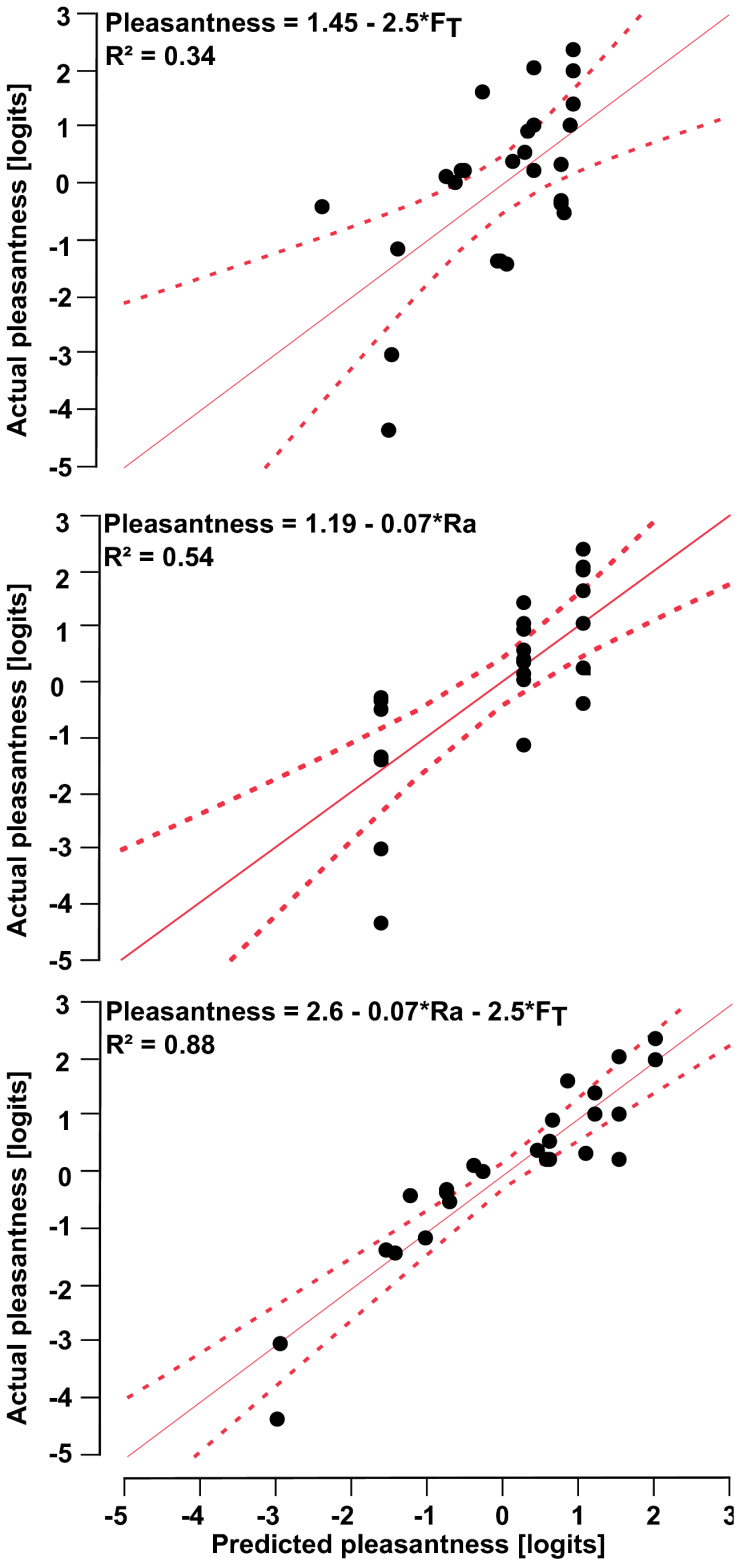
Illustration of the multiple regression analysis models. Panels illustrates the actual versus the predicted pleasantness levels if the predictor variables are the average tangential force (*F_T_*) alone (top), average roughness level (*Ra*) alone (middle), or both *F_T_* and *Ra* (bottom). Dotted lines delimit the 95% confidence interval.

To determine whether we could predict the linear and unidimensional pleasantness measures of the 26 stimuli of the *Passive Pleasant Touch Scale*, we performed linear multiple regression analyses. The pleasantness of the stimulus was defined as the dependent variable, and *F_T_*, *F_N_*, *Ra*, *T_S_*, *M*, and *T_F_* were independent variables. *Ra* and *F_T_* significantly predicted 88% of the variance of the pleasantness measures, with *Ra* predicting a greater portion (54%) than *F_T_* (34%). Identical results were found through a second regression analysis, in which pleasantness was defined as the dependent variable, but only *Ra* and *F_T_* were used as independent ones (Figure 4). Panels of Figure 4 show the actual pleasantness measures versus the expected ones if only *F_T_* (top), only *Ra* (middle), or both *F_T_* and *Ra* (bottom) are used to predict pleasantness. The three equations on this figure indicate all that surfaces were perceived as less pleasant when (i) their *Ra* increased and/or (ii) their *F_T_* increased.

**Figure 5:**
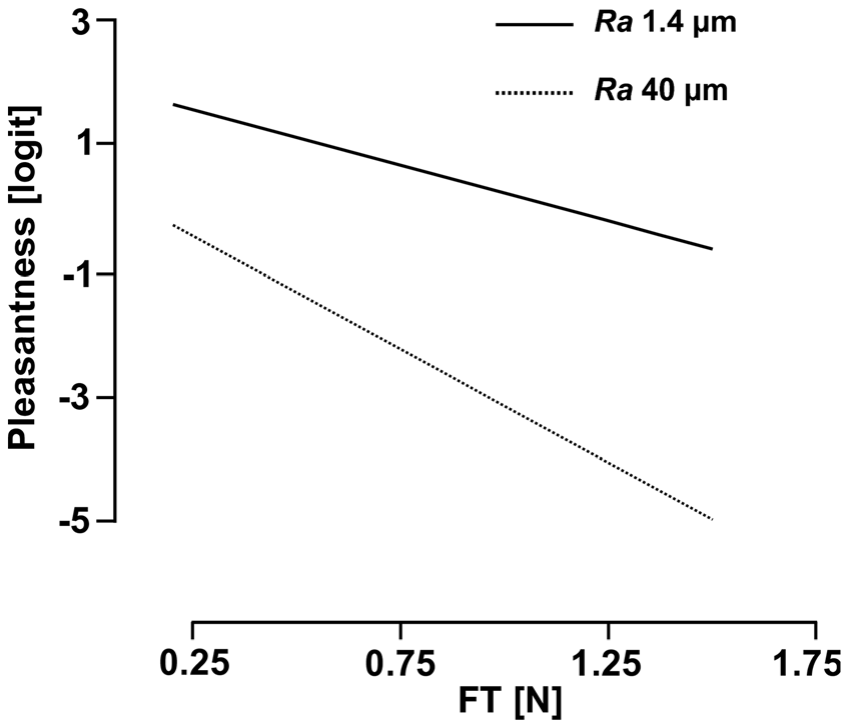
Illustration of the interaction effect between *Ra* and *F_T_*.

Finally, when taking into account the interaction between *Ra* and *F_T_* (i.e. Ra*F_T_) a total 91% of the variance of the pleasantness measures were predicted. Thus, this interaction accounted for 3% of the variance in pleasantness. The interaction effect is illustrated in Figure 5, which shows that the smooth plate was always perceived as more pleasant than the rough plate, and that pleasantness was negatively correlated with *F_T_* in both cases. However, this latter correlation depended on *Ra*; the higher *F_T_*, the more the rough plate will induce higher unpleasant perceptions compared to those induced by the smooth one. Table 3 summarizes the results of these regression analyses.

**Table 3:** Regression analyses.

	Dependent	Independent	Significant		
Analysis	variable	variables	variables	Total R^2^	R^2^ per variable
1	pleasantness	*F_N_, F_T_, Ra, T_S_, T_F_, M*	*F_T_, Ra*	0.88	R^2^ *_Ra_* = 0.54
					R^2^ *_FT_* = 0.34
2	pleasantness	*F_T_, Ra*	*F_T_, Ra*	0.88	R^2^ *_Ra_* = 0.54
					R^2^ *_FT_* = 0.34
3	pleasantness	F_T_, Ra, F_T_*Ra	F_T_, Ra, F_T_*Ra	0.91	R^2^ _Ra*FT_ = 0.91

*F_N_*: normal force; *F_T_*: tangential force; *Ra*: average roughness level; *T_S_*: stimulus temperature; *T_F_*: fingertip temperature; *M*: fingertip moisture level; F_T_*Ra: interaction of *F_T_* and *Ra*.

**Figure 6:**
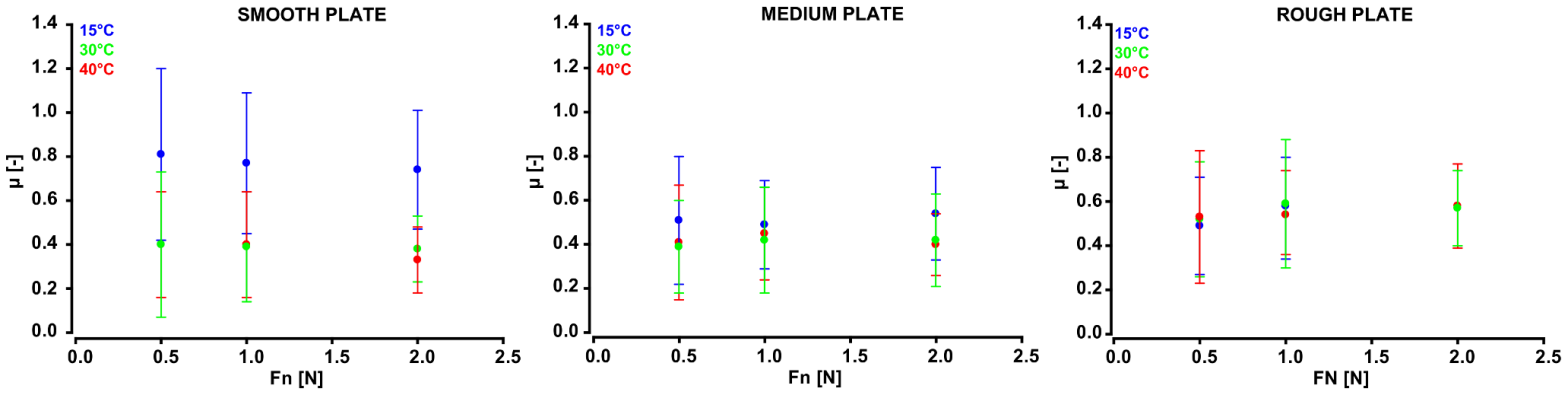
Illustration of the correlation between *μ* and *F_N_*. From left to right, this correlation is shown for the smooth, medium-roughness, and rough plates. Each point represents the mean ± standard deviation value of *μ*, according to the three temperature levels.

Although the above analyses indicated that *T_S_* had no significant direct influence on the pleasantness, we investigated whether *T_S_* had an impact on *μ*. The RM-ANOVA results indicated that *T_S_* seemed to have an impact on *μ* for the smooth and medium-roughness plates only (Figure 6 and Table 4). Multiple comparisons (Bonferroni) highlighted that, regardless of *F_N_*, *μ* was significantly higher if the smooth plate was applied at 15°C than at 30°C or 40°C. When the medium-roughness plate was applied at 0.5 or 2N, *μ* was significantly higher in the 15°C condition compared to the 30°C or 40°C condition. The effect of *T_S_* on *μ* was smaller during stimulation with the medium-roughness plate compared to stimulation with the smooth plate, indicating that *Ra* also had an impact on *μ* (*see* Table 4).

**Table 4:** RM-ANOVA investigating in the effect of the *T_S_* on *μ*.

	RM-ANOVA	RM-ANOVA	RM-ANOVA
Plate	(plate applied at 0.5 N)	(plate applied at 1 N)	(plate applied at 2 N)
Smooth	F_2,42_ = 16.92; p < 0.001	F_2,42_ = 25.20; p < 0.001	F_2,42_ = 67.25; p < 0.001
Medium	F_2,42_ = 8.24; p = 0.001	F_2,42_ = 1.46; p = 0.24	F_2,42_ = 17.1; p < 0.001
Rough	F_2,42_ = 0.90; p = 0.41	F_2,42_ = 2.38; p = 0.11	F_2,42_ = 1.23; p = 0.30

*RM-ANOVA*: repeated-measure analysis of variance.

## Discussion

We have described the influence of physical factors on the pleasantness perception during *passive* fingertip stimulation. The average roughness and average tangential force at the finger-surface interface were important factors influencing the perception of pleasantness. The range of stimulus temperatures and normal forces used in this study did not allow us to highlight any direct correlation between pleasantness and stimulus temperature, or between pleasantness and the average normal force.

Passive fingertip stimulation was a relevant experimental procedure for several reasons. First, this stimulation procedure allowed the study of an intentional change in normal force on pleasantness perception. Indeed, a past active touch study [25] highlighted that subjects do not significantly adapt their spontaneous exploration normal force. As a consequence, the effect of this variable could not be investigated in that past study. Second, as the experimental device was equipped with thermal stimulation modules, the effect of surface temperature on pleasantness perception could be investigated. Third, through this study, we could compare factors being involved in the pleasantness perception resulting from an active surface exploration to those being implicated in the pleasantness perception during passive fingertip stimulation. These points will be discussed hereafter.

The results of this study showed that the perceived pleasantness was not related to the *normal force* when this parameter was varied between 0.5 and 2 N. Nevertheless, having shown that the tangential force is negatively correlated with pleasantness, it can be hypothesized that the normal force has an indirect effect on pleasantness through its influence on the tangential force. Consider, for example, the application of the same stimulus to the fingertip at different levels of normal force. Although the net increase/decrease in load may not seem to alter the perception of pleasantness, the change in normal force alters the tangential force, which, in turn, leads to changes in pleasantness perception. Moreover, participants have been demonstrated to prefer certain exploration strategies, regardless of the surface being explored [25], [29]. Taken together, these results suggest that participants rate pleasantness levels, amongst others, by comparing the friction forces that arise during surface exploration.

The *innocuous thermal variations* of the different stimuli did not directly influence the pleasantness measures. However, regardless of the normal force, the coefficient of dynamic friction for the smooth plate was higher at 15°C than at 30°C or 40°C. This effect of temperature on friction was not observed on the rough plate (Figure 6 and Table 4). Our observation is supported by the equation proposed by Van Kuilenburg et al. [30], in which the dynamic coefficient of friction is linked to the temperature difference between the finger and the surface (*ΔT*). These variables are positively correlated in that equation, indicating that higher temperature differences will induce higher friction values. Our subjects had an average fingertip temperature of 32.9°C ± 2.4°C. Consequently, the temperature difference was higher for stimuli applied at 15°C (mean *ΔT* of 17.9°C) than for those applied at 30°C (mean *ΔT* of 2.9°C) or at 40°C (mean *ΔT* of 7.1°C). It can be hypothesized that the fingertip moisture evaporates more quickly on stimuli at 30°C or 40°C than on those at 15°C. Furthermore, increasing the fingertip moisture level has the potential to increase the dynamic coefficient of friction at the finger-surface interface during stimulation [31]–[34]. Therefore, a potential explanation for the friction increase observed in the 15°C stimulation condition could be that higher moisture contents were present at the surfaces of the stimuli. Interestingly, the heat transfer rate is larger for smooth than for rough surfaces, likely because smooth surfaces offer a larger heat exchange area to the skin [30]. Taken together, these facts could explain why: (i) the dynamic coefficient of friction was higher when the smooth plate was applied at 15°C, and (ii) the stimulus temperature had no effect on the dynamic coefficient of friction resulting from stimulations with the rough plate.

Results of this study and our past ones [24]–[25] indicate that the factors important for pleasantness perception during *active touch*, such as the average roughness (or surface topography) and the average tangential force, are similar to those dictating pleasantness perceptions during *passive fingertip* stimulation. Nevertheless, the literature is unclear as to whether active and passive touch yield similar perceptual performances. Some studies have indicated that active object/surface exploration induces different perceptions compared to the passive exploration of an identical object/surface [35]–[38]. Others have shown that both strategies yield similar perceptual performances [39]–[40], or that reducing behavioural differences between active and passive exploration strategies reduces differences in perceptual performance [41].

The pleasantness level of one stimulus (rough plate at 2N and 15°C) could not be estimated accurately since this stimulus was extremely less pleasant than other stimuli as all the subjects rated it as “*unpleasant*”. This result is not surprising, as the multiple regression analysis highlighted that *Ra* and *F_T_* were negatively linked to pleasantness. This stimulus combined thus both high *F_T_* (see Table 1) and high *Ra*. Even if we could not find a systematic link between pleasantness and surface temperature, it seems that if a high *Ra* is combined with a high *F_T_*, low temperature might make a surface even more unpleasant.

In the present study, stimuli were all applied with a same velocity. Although the stimulation velocity has little effect on the sensation of roughness [42], the sliding velocity influences the friction induced during fingertip stimulation [33] and the spectral content of tactile cues [43]. As our findings indicate that the perception of pleasantness is influenced by the surface roughness and friction during stimulation, it could be of interest to test the effect of the stimulation velocity on the pleasantness perception.

A previous study suggested that the fingertip moisture level may influence pleasantness perception during *active* surface exploration with the fingertips [24]. That previous investigation was possible owing to the very large range of fingertip moisture levels of participants [24]. In the present study, all of the participants had relatively low moisture levels; thus, we were unable to directly investigate the link between fingertip moisture level and pleasantness perception. Future studies could specifically address the impact of fingertip moisture on pleasantness perception induced through passive fingertip stimulation.

In the present study none of the subjects had a specific touch-related occupation (*e.g.* carpenter or brick layer). As a consequence, it was not possible in this study to investigate whether such occupations might have had an impact on the pleasantness perception. It could therefore be of interest to specifically recruit, in a future study, participants having a touch-related occupation as well as age and gender matched participants which do not have a touch-related occupation. Through an invariance analysis, the Rasch model analysis would allow to highlight whether this factor has a significant effect on the pleasantness perception.

Though CT-afferents are missing from glabrous skin sites, such as the fingertips [13]–[15], the present study as well as past ones [24], [25] highlight that pleasantness perception is present and even quantifiable at fingertip level. A study comparing ratings of pleasantness arising from stimulation of hairy versus glabrous skin sites indicate that stroking the hairy skin site arose greater affective values than those induced by the stimulation of a glabrous skin site [44]. Altogether, these findings point thus to the fact that pleasantness, as the other dimensions of tactile perception, arises from the integration of a complex variety of information originating peripherally from the various receptor families distributed across the skin. Based on our results, it seems that mechanoreceptors being implicated in the detection and transmission of surface topography as well as tangential forces are important for this perception in addition to CT-afferents. It is therefore most plausible that not one single afferent fiber system is responsible for the perception and transmission of pleasant stimuli applied to the glabrous skin of the fingertip, but that this transmission can be effectuated through the combined activity of the several afferent systems. The PC and SAI channels might both be implicated in the perception of pleasantness as they have been described to be active during surface exploration having a coarse (SAI) and fine (PC) textured surface [45], [46]. Furthermore, SAI and RA are potentially involved in the perception of pleasantness as they were described to “*provide the neural basis for peripheral signals of tangential force magnitude*” [47].

## Supporting Information

Introduction S1 **Introduction to the Rasch model.**
(DOCX)
