# Supplementary material for: Physical Factors Influencing Pleasant Touch during Passive Fingertip Stimulation
Source: PLoS One. 2014 Jul 7;9(7):e101361. doi: 10.1371/journal.pone.0101361 (PMC4084823; doi:10.1371/journal.pone.0101361)
Supplement: Introduction S1 — Introduction to the Rasch model. (DOCX) [file pone.0101361.s001.docx]

**Supporting Information – Introduction S1**

*The Rasch Model*

Measurement requires an abstraction or construct which represents the attribute being measured. Indeed, when measuring, for example, the length of an object, we refer to an abstract or latent continuum represented on the measurement instrument being used. The measure is generally conceived as a point along the abstract measurement scale, which is implemented by the instrument. By analogy, to measure pleasantness, we should refer to an abstract or latent continuum of pleasantness. Measuring the pleasantness of a surface should thus be equivalent to determining the position of that surface along an underlying scale of pleasantness. A line conceptualized from “less pleasant” to “more pleasant” should represent a gradient and increasing levels of pleasantness could be materialized by surfaces of increasing pleasantness. In general, a scale requires thus a manifest order and should focus on one attribute at a time.

To measure a latent variable (*i.e.* variables which are not directly observable), such as the pleasantness of a surface, an experimental procedure is required to observe how much pleasantness is concealed within the surface. This is typically achieved by using, for example, a questionnaire or a set of stimuli being rated by using either categorical rating or magnitude estimation methods. For instance, to measure the pleasantness of a surface through categorical rating, subjects’ perception of pleasantness can be rated using a dichotomous response format, *e.g.* “unpleasant” (scored 0) and “pleasant” (scored 1), or a polytomous response format, *e.g.* “unpleasant” (scored 0), “pleasant” (scored 1) and “very pleasant” (scored 2). Usually, the response scores will be summed together into a total score. Such measurement provides thus *ordinal scores* without unit. Ordinal scores come, however, with severe shortcomings, precluding the *objective measurement^[[1]](#footnote-1)^* of a variable [1-7]. Firstly, ordinal scores are not necessarily *linear* (*i.e.* the measurement unit is not constant throughout the measurement range, [8]). For instance, equal distances (*e.g.* from 0 to 1 and from 1 to 2) may not reflect the same amount of the measured variable. Secondly, ordinal scores are not necessarily *unidimensional* (*i.e.* the score reflects not only one attribute of the subject, [8]). As a result, adding individual ordinal scores may result in a total score that is meaningless [9]. Thirdly, ordinal scores are not necessarily *objective* [8]. For example, a given subject taking an easy test will get a high score, while he/she will get a lower score when taking a more difficult test of the same kind. Hence, the difficulty of the test needs to be extracted from the score before appraising the subject’s measure [10]. Lastly, ordinal scores are not necessarily *invariant* under changes of the populations employed to generate the data (*e.g.* systematic differences between males/females) [8]. In contrast to categorical rating, magnitude estimation is an unlimited rating procedure where respondents can freely choose a number reflecting their pleasantness perception [11]. Magnitude estimation can either be referenced to a standard (*i.e.* reference-standard magnitude estimation) or be absolute (*i.e.* absolute magnitude estimation). This rating procedure was originally constructed to overcome the shortcomings of measurement methods generating ordinal data. However, studies question whether this aim was really achieved and even state that, similarly to categorical rating, magnitude estimation yields ordinal scores [1, 5].

The Rasch model can be used to establish linear, unidimensional, and invariant scales from ordinal scores by locating subjects and items (*i.e.* surfaces to be perceived) along a single underlying scale [2, 8-9]. Indeed, the formulation of the Rasch model is articulated around the requirements of unidimensionality and invariance. The model requires unidimensionality in the data since only one trait of the subject and one trait of the item are modelled to determine the probability of the observed response when any subject answers any item. For polytomous response scales, an additional parameter is used to model the threshold between successive response categories. The model also requires invariance in the subject/item interaction since the probability of observing a given response category is modelled to vary neither with person factors (*e.g.* age, gender, occupation) other than the one being measured nor with item factors (*e.g.* surface hardness or water retention) other than the one being measured. Both of these qualities are required for any fundamental measurement [2, 6-10]. For instance, the length of an object can be measured with a meter stick, which is supposed to provide results that are independent of the object’s color, shape or temperature (*i.e.* length measurement instruments are used as if they were unidimensional). When comparing the measure of two objects obtained with the same ruler the measures are not supposed to depend on objects properties (*e.g.* shape and color) other than the length being measured (*i.e.* length measurement instruments are used as if they were invariant relative to objects qualities other than their length). Furthermore, the Rasch model requires an independence of the object’s measure relative to the instrument (*i.e.* the items) which was used to obtain the measure. Provided that all items fit with the same latent scale the resulting measure should not depend on the particular items that were used to obtain it. This may be illustrated by the measurement of an object’s length, which can be equally measured with a ruler, a meter tape or a folding meter as all these instruments refer to the same underlying definition of the meter (the unit of the latent scale underlying all length instruments).

Unlike the scores obtained by any traditional rating method, the Rasch model provides measures that are expressed on a linear scale. The unit on the measurement scale is the logit (which stands for log-odds-unit) computed as the logarithm of the odds for subject “*n*” to pass threshold “*k*” on item “*i*”:

$$\ln\left( \frac{P_{nik}}{P_{nik-1}} \right)=\beta_{n}-\delta_{i}-\tau_{k}$$

where *β_n_* is the subject leniency (*i.e.* “pleasantness satisfaction level”), *δ_i_* is the surface pleasantness, *τ_k_* is the threshold between response categories k and k-1, *P_nik_* is the probability for subject “n” to select category “k” for item “i” and *P_nik-1_* is the probability for subject “n” to select category “k-1” for item “i”. The resulting measurement scale is linear since, whatever the stimulus, an increase of the subject location by one logit corresponds to an increase in the odds for the subject to perceive the stimulus as “pleasant” rather than “unpleasant” by a constant factor of 2.71 (*i.e.* the Neperian constant) [12]. Just like one centimeter represents the same amount of length throughout the range of any length measurement instrument.

Finally, it is interesting to note that Koskey et al. (2013) [13] used the Rasch model to compare the rating scale functioning of categorical rating to that of magnitude estimation. In their study, each participant had to complete two versions, one categorical rating and one magnitude estimation, of a “*Survey of Research Attitudes*” (SRA). The analyses showed that the overall scale structure of the categorical rating scale was more stable compared to the magnitude estimation scale. Indeed, in contrast to categorical rating, the step calibrations of the magnitude estimation were disordered switching back and forth between positive and negative thresholds. This indicated that participants were not able to use magnitude estimation scales in a monotonic and quantitatively meaningful way [13]. Consequently, even if subjects reported a numeric range to the SRA, the meaning of these numbers remained unknown, which made any interpretation difficult [13]. Moreover, it was detected that a majority of subjects preferred the categorical rating method over magnitude estimation.

The here above observations point to fact that, independently of the scaling method used to rate a variable, fundamental scaling properties (*i.e.* unidimensionality, linearity and invariance) should be assessed by using modern psychometric methods (*e.g.* the Rasch model) prior to further interpretation of the data.

*References*

1 Mellers BA (1983) Evidence against "absolute" scaling. Percept Psychophys 33: 523-526.

2 Rasch G (1960) Probabilistic models for some intelligence and attainment tests. Chicago: Mesa Press 199 p.

3 Tesio L, Simone A, Bernardinello M (2007) Rehabilitation and outcome measurement: where is Rasch analysis-going? Eura Medicophys 43:417-426.

4 Thurstone LL (1928) Attitudes can be measured. American Journal of Sociology 33: 529-554.

5 Wills CE, Moore CF (1994) A controversy in scaling of subjective states: magnitude estimation versus category rating methods. Res Nurs Health 17: 231-237.

6 Merbitz C, Morris J, Grip JC (1989) Ordinal scales and foundations of misinference. Arch Phys Med Rehabil 70: 308-312.

7 Penta M, Arnould C, Decruynaere C (2005) Développer et interpréter une échelle de mesure. Applications du modèle de Rasch. Sprimont: Mardaga 186 p.

8 Bond TG, Fox CM (2001) Applying The Rasch Model: Fundamental Measurement in the Human Sciences. Mahwah, New Jersey: Lawrence Erlbaum Associates 255 p.

9 Wright BD, Linacre JM (1989) Observations are Always Ordinal; Measurements, however, Must be Interval. Arch Phys Med Rehabil 70: 857-860.

10 Hobart J, Cano S (2009) Improving the evaluation of therapeutic interventions in multiple sclerosis: the role of new psychometric methods. Health Technol Assess 13: 1-177.

11 Stevens SS (1975) Psychophysics: Introduction to its perceptual, neural, and social prospects. New Jersey: John Wiley & Sons.

12 Wright BD, Masters GN (1982) Rating Scale Analysis. Rasch Measurement. Chicago: Mesa Press 206 p.

13 Koskey KL, Sondergeld TA, Beltyukova SA, Fox CM (2013) An experimental study using Rasch analysis to compare absolute magnitude estimation and categorical rating scaling as applied in survey research. J Appl Meas 14: 262-281.

1. The Institute for Objective Measurement (IOM, www. Rasch.org) defines objective measurement as “*the repetition of a unit amount that maintains its size, within an allowable range of error, no matter which instrument, intended to measure the variable of interest, is used and no matter who or what relevant person or thing is measured*”. [↑](#footnote-ref-1)
